# A boy with Prader-Willi syndrome: unmasking precocious puberty during growth hormone replacement therapy

**DOI:** 10.1590/2359-3997000000196

**Published:** 2016-08-23

**Authors:** Natasha G. Ludwig, Rafael F. Radaeli, Mariana M. X. Silva, Camila M. Romero, Alexandre J. F. Carrilho, Danielle Bessa, Delanie B. Macedo, Maria L. Oliveira, Ana Claudia Latronico, Tânia L. Mazzuco

**Affiliations:** 1 Centro de Ciências da Saúde Universidade Estadual de Londrina Londrina PR Brasil Pós-Graduação em Ciências da Saúde, Centro de Ciências da Saúde, Universidade Estadual de Londrina (UEL), Londrina, PR, Brasil; 2 Hospital Universitário UEL Londrina PR Brasil Serviço de Endocrinologia do Hospital Universitário, UEL, Londrina, PR, Brasil; 3 Hospital das Clínicas Faculdade de Medicina Universidade de São Paulo São Paulo SP Brasil Laboratório de Hormônios e Genética Molecular (LIM/42), Hospital das Clínicas, Faculdade de Medicina da Universidade de São Paulo (HC-FMUSP), São Paulo, SP, Brasil

## Abstract

Prader-Willi syndrome (PWS) is a genetic disorder frequently characterized by obesity, growth hormone deficiency, genital abnormalities, and hypogonadotropic hypogonadism. Incomplete or delayed pubertal development as well as premature adrenarche are usually found in PWS, whereas central precocious puberty (CPP) is very rare. This study aimed to report the clinical and biochemical follow-up of a PWS boy with CPP and to discuss the management of pubertal growth. By the age of 6, he had obesity, short stature, and many clinical criteria of PWS diagnosis, which was confirmed by DNA methylation test. Therapy with recombinant human growth hormone (rhGH) replacement (0.15 IU/kg/day) was started. Later, he presented psychomotor agitation, aggressive behavior, and increased testicular volume. Laboratory analyses were consistent with the diagnosis of CPP (gonadorelin-stimulated LH peak 15.8 IU/L, testosterone 54.7 ng/dL). The patient was then treated with gonadotropin-releasing hormone analog (GnRHa). Hypothalamic dysfunctions have been implicated in hormonal disturbances related to pubertal development, but no morphologic abnormalities were detected in the present case. Additional methylation analysis (MS-MLPA) of the chromosome 15q11 locus confirmed PWS diagnosis. We presented the fifth case of CPP in a genetically-confirmed PWS male. Combined therapy with GnRHa and rhGH may be beneficial in this rare condition of precocious pubertal development in PWS.

## INTRODUCTION

Prader-Willi syndrome (PWS), also known as Prader-Labhart-Willi syndrome, is a complex neurogenetic disorder characterized by neonatal hypotonia, psychomotor delay, early-onset hyperphagia, short stature, hypogonadism, sleep disturbance, learning disabilities, and behavioral and psychiatric disorders; it also represents the most common form of genetic obesity. Its incidence is estimated between 1:10,000 and 1:30,000 living births with the prevalence of 1:50,000. The expression of paternally active genes located on chromosome 15q11-q13 is lost in PWS, which may occur due to a deletion in this chromosomal segment (65-70%), maternal uniparental dissomy (25-30%), or imprinting defects (1-2%), even though rare gene mutation (< 0.1%) and balanced translocation (0.1%) can also be found (1,2). Diagnosis of PWS is made according to the Holm and Cassidy criteria (3), but it must be confirmed by genetic analysis.

Patients with PWS have multiple characteristics associated with hypothalamic dysfunctions, such as hyperphagia, growth hormone (GH) deficiency, and abnormal pubertal development (1). Usually these patients have hypogonadism manifested as genital hypoplasia and unilateral or bilateral cryptorchidism, incomplete pubertal development, and infertility, which are attributed to both hypothalamic dysfunction and/or to primary gonadal defect (4). In fact, hypogonadism is one of the eight major clinical diagnostic signs of PWS, whereas isolated premature adrenarche is frequently observed and is accepted as a minor criterion (3).

Despite the common clinical picture of delayed or incomplete puberty, rare cases with true precocious puberty have been described in this syndrome (5). Here, we report a male patient with PWS caused by a classical hypermethylation of the *locus* 15q11 who experienced growth hormone deficiency and central precocious puberty (CPP). To the best of our knowledge, this is the first case of CPP reported in boys with PWS during GH replacement. Informed consent was obtained from the patient’s legal guardian for publishing this case report.

## CASE REPORT

**Figure 1:**
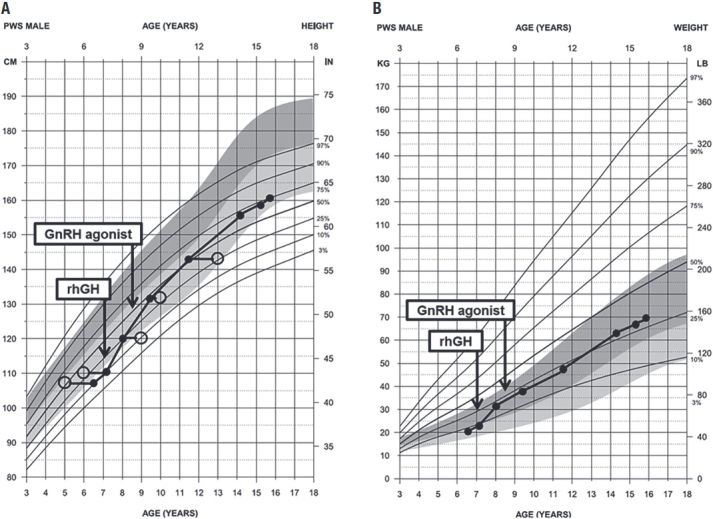
Growth and obesity status of the reported patient: a boy with Prader-Willi syndrome. Stature (A) and weight (B) are plotted against age as solid circles in syndrome-specific growth standards. Bone ages are represented as open circles (A). Arrows indicate the beginning of the growth hormone replacement (rhGH, somatropin) and the depot leuprorelin acetate therapy (GnRHa) for central precocious puberty. The growth curves were constructed using data from the PWS-specific growth chart (6).

The patient was born at term by operative delivery at 39 gestational weeks (2.46 kg, 47 cm). His parents were healthy, non-consanguineous, and from Caucasian descent. Hypotonia and bilateral cryptorchidism were present at birth as well as poor sucking and low weight gain during the first month of life. Failure to thrive was diagnosed in the next months; no brain abnormalities were detected on the computed tomography scan. At the age of 2, he began to develop hyperphagia, followed by rapid weight gain and signs of mild psychomotor deficiencies; the clinical diagnosis of PWS was then first suspected. At the age of 3, he was submitted to surgical orchiopexy. At the age of 6, he was referred to our pediatric endocrinology unit for evaluation of obesity and short stature. His height was below the 3^rd^ percentile, weight between the 25-50^th^ percentile range, body mass index between the 90-95^th^ percentile range with normal bone density and 37.9% total body fat. At presentation, he had no signs of sexual development (Tanner stage I): no pubic hair, nonpalpable left testis (1.05 cm^3^), normal right testis (1.53 cm^3^) (both measured by ultrasonography), and stretched penile length of 1.5 cm. Otherwise, his examination was normal, except for low ears implantation, relatively small hands and feet, valgus knee, and mild learning disabilities. The Holm and Cassidy criteria score was 9; the genetic test demonstrated only the methylated maternal allele, thus confirming the clinical diagnosis of PWS by the methylation-specific PCR (MS-PCR) performed ten years ago. Figure 1 presents anthropometric measurements plotted on current standardized growth curves for PWS subjects (6).

Initial laboratory analyses resulted in prepubertal hormone levels (Table 1). The peak growth hormone response to clonidine testing was 1.75 µg/L (normal > 7 µg/L), suggesting the diagnosis of GH deficiency. Considering that the patient presented an inadequate growth rate but had no nutritional deficiency or hypothyroidism, therapy with recombinant human growth hormone (rhGH) replacement (0.15 IU/kg/day) was started at the age of 7 (Figure 1). In the beginning of the rhGH treatment, his insulin-like growth factor 1 (IGF-1) level recovered to the middle-to-upper normal range (Table 1), and his growth velocity was 6 cm/10 months.

**Table 1:** Nine-year follow-up of anthropometric evaluation, clinical examination, and laboratory findings of a Prader-Willi syndrome boy with central precocious puberty

Anthropometrical data																				
Age (years)	6.60		7.10		8.00			8.50			8.80		9.40		10.80			11.50	14.30	15.20
Height (cm) [z-score]^a^	107 [-2.32]		110 [-2.37]		120.50 [-1.20]			123 [-1.19]			126 [-0.82]		132 [-0.45]		138 [-0.63]			143 [-0.43]	156 [-1.20]	159 [-1.38]
Weight (kg)	20.9		23.2		30.9			32.9			33.4		38.3		45			48.8	62.3	66
BMI (kg/m^2^) [centile]^a^	18.25 [95]		19.17 [97]		21.30 [99]			21.6 [99]			21.04 [98]		21.90 [98]		23.60 [99]			23.80 [98]	25.50 [97]	26.10 [96]

**Growth therapy follow-up**																				

IGF-1^b^ (µg/L)	75.40				339			518					346					466	218	
[age-specific reference ranges]	[52-297]				[57-316]			[64-345]					[74-388]					[111-551]	[220-972]	
rhGH (U/kg/day)			Somatropin (0.15)								Somatropin (0.1)									

**Puberty blocking follow-up**																				

Bone age^c^ (years)	5	6		9										10			13		14	
Genitalia stage (Tanner)	I	I		II					II			II		II		II-III	III		III	IV
Right testis volume^d^ (mL)		3		4					4								3		3	6
Left testis volume^d^ (mL)	np	np		2 (r)					2.5								np		np	5
Pubic hair (Tanner)	I	I		II					II-III					III-IV		III-IV	III		III	IV
LH (IU/L)	< 0.50			0.50					0.47					1.66		0.94			< 0.07	
FSH (IU/L)	2.88			5.60^e^					8.80^e^					8.70		5.85			0.15	
Testosterone (ng/dL)	< 0.1			13^e^					55^e^					19		< 20			14.45	
GnRHa (mg/month)										Leuprorelin (3.75)										

^a^ STAT GrowthCharts^Tm^ (WHO, Geneva, 2006). ^b^ IGF-I measured by an automated chemiluminescence immunoassay (Nichols Institute Diagnostics, San Clemente, CA, USA). ^c^ According to the Greulich and Pyle method. ^d^ Measured using Prader orchidometer. ^e^ Hormone levels above the normal range.

BMI: body mass index; FSH: follicle-stimulating hormone; GnRHa: gonadotropin-releasing hormone analog; IGF-1: insulin-like growth factor 1; LH: luteinizing hormone; np: nonpalpable; r: retractile; rhGH: recombinant human growth hormone.

At the age of 7.6, his parents reported he was presenting psychomotor agitation, aggressive behavior, and also frequent penile erections. Physical examination revealed signs of puberty, including asymmetric enlargement of testes (1.5 and 4 cm^3^, left and right respectively) and penis length (4.5 cm). Biochemical and imaging evaluation for peripheral precocious puberty revealed no evidence of congenital adrenal hyperplasia, human chorionic gonadotropin (hCG), or androgen-secreting tumors. At the age of 8 (Table 1), he presented Tanner stage II, bone age of a 9-year old, and laboratory analyses confirmed the diagnosis of CPP: precocious pubertal level of total testosterone (54.7 ng/dL) and luteinizing hormone (LH) peak 15.8 IU/L after gonadotropin-releasing hormone (GnRH) stimulation test; magnetic resonance imaging (MRI) of hypothalamic-pituitary region was normal.

Treatment for precocious puberty was started at the age of 8.8 with GnRH analog (GnRHa) (depot leuprorelin acetate 3.75 mg i.m. every 28 days), and the rhGH dose was adjusted to 0.1 IU/kg/day. Patient’s response to treatment was satisfactory, as evaluated under pubertal blockade by GnRHa (Table 1) at the age of 9.4: total testosterone was 18.7 ng/dL (normal for age 9.8 – 19.6 ng/dL), LH two hours after leuprorelin 3.75 mg i.m. was 1.66 IU/mL, and IGF-1 was 346 µg/L (normal for age 74-388 µg/L). He was treated with leuprorelin 11.25 mg i.m. every three months until the age of 13. Bone age deviation and pubertal Tanner stage III had no progression during GnRHa therapy, with spontaneous resolution of testicular size discrepancy, but a second left orchiopexy was needed. At the last endocrine visit, Tanner stage was V, growth velocity was adequate for age, and his treatment was rhGH 0.08 IU/kg/day and topiramate 50 mg twice a day. We have recently performed a more precise molecular diagnosis test, the methylation-specific multiplex ligation-dependent probe amplification (MS-MLPA) of the locus 15q11 performed by SALSA MS-MLPA (Kit ME028, MRC Holland, Amsterdam, Netherlands). MS-MLPA revealed a hypermethylation pattern of the SNPRN probes, suggesting that the two chromosome 15s came from the mother and none from the father (maternal uniparental disomy) or that a maternal-only DNA-methylation pattern occurred despite the biparental inheritance (imprinting defect). No abnormality in copy number was detected in this region, excluding a deletion.

## DISCUSSION

Here, we described the case of a boy presenting common features of PWS including obesity, short stature, hypotonia, and hypogonadism (characterized by cryptorchidism and small penis); after the onset of rhGH therapy, he surprisingly presented early signs of pubertal development. According to a large database of PWS children, growth hormone deficiency was present in 80% of patients, and 86.7% were treated; hypogonadism was present in 49% of patients (7). Gonadal axis function, however, is not homogeneous; normal gonadotropin response to GnRH test is quite common. but hypergonadotropic hypogonadism has also been described (4). Moreover, many children present premature adrenarche that is manifested with slightly advanced bone maturation related to obesity; usually, puberty fails to progress beyond this stage. Nevertheless, CPP has been rarely reported in PWS (5). Our patient fulfilled all diagnostic criteria for CPP (i.e., age, advanced skeletal age, testicular volume, pubertal LH response to GnRH test, and androgen levels). According to our literature review, this is the fifth case of CPP in a genetically-confirmed PWS male (5,8-10). GnRH blockade has been reported in two cases (5,9). Abnormal imaging of the hypothalamic-pituitary region was observed in two cases: empty sella (8) and pituitary microadenoma (9).

The locus 15q11 contains a domain of imprinted genes in which loss of expression of the paternally-inherited allele of genes including *SNRPN*, *NDN*, *MAGEL2*, and *MKRN3* is associated with PWS. Mapping of microdeletions associated with PWS has identified a 4.3 kb region of the *SNPRN* gene that appears to be required for establishment and/or maintenance of the paternal epigenotype across the imprinting center domain (11). Therefore, it has been speculated that deleterious defects such as paternal deletion, maternal uniparental disomy, and methylation abnormalities of the SNPRN probes in the MS-MLPA could lead to the loss of paternal PWS-imprinting center and presumably lose expression of the distal genes, including the makorin ring finger protein 3 (*MKRN3*) (12). In this study, we demonstrated a classical hypermethylation pattern of 4 SNPRN probes, indicating a PWS diagnosis (1). Interestingly, several loss-of-functions mutations of *MKRN3* gene, an imprinted gene located on chromosome 15q11, Prader-Willi critical region, were described in non-syndromic patients with familial CPP (13,14). However, the MKRN3 role in the PWS associated with CPP remains unknown. Here, the genetic diagnosis of PWS was first detected using a DNA methylation analysis and then confirmed by a MS-MLPA. The DNA methylation is usually the first line test in the PWS diagnosis and can correctly diagnose PWS in up to 99% of the cases; however, this technique cannot distinguish between deletions, uniparental disomy, or imprinting defects (1). The advantage of MS-MLPA over traditional DNA methylation is that MS-MLPA investigates five distinct differentially methylated sites (4 probes for SNPRN and one probe for NDN methylated sites) rather than just one locus and can assess the deletion status at the same time as the DNA methylation

The natural course of PWS is a significant increase in fat mass over the years that can be worsened by GH deficiency (2). Beyond improving longitudinal growth, the rhGH replacement in our patient also aimed to improve body composition, bone density, physical capacity, basal energy consumption, cognition, and life quality. As we could observe, there was a significant increase in longitudinal growth rate (10 cm/year) in the first year of treatment with rhGH, which is uncommon in patients with PWS, once they usually have low IGF-1 levels compared to exogenous obesity. GH secretion and puberty may be correlated since GH exerts direct effects on gonadal function and may influence reproductive activity by increasing secretion and sensitivity of gonadotropin releasing hormone (15). However, during the rhGH therapy in PWS children, the simultaneous activation of the pituitary-gonadal axis is certainly very unusual (7). Another case of male PWS previously reported with CPP was treated by replacing rhGH, but his pubertal development had preceded rhGH therapy (5).

Despite the fact that precocious puberty has been recognized in few PWS cases, there is controversy whether treatment with GnRHa should be used. Patients with PWS commonly fail to complete puberty, and, as a consequence, there was no final stature loss, in spite of slight bone age advance (4). In the present case, the decision to treat with GnRHa was made because of the development of aggressive behavior but also the bone age advance. The GnRHa was started at the age of 9 and showed good clinical and laboratory response. After 6 months of treatment, adequate puberty blocking was evidenced by normal bone age progression and stabilization of secondary sexual characteristics.

The rare manifestations of CPP in patients with PWS have been attributed to brain lesions (5,10). Moreover, morphological abnormalities were also associated with GH deficiency in PWS patients, and hypothalamic dysfunction was frequently associated with pituitary hypoplasia (10). Conversely to these observations, our patient’s imaging of the hypothalamic-pituitary region was normal. In some conditions, CPP could be associated with rhGH exposure during childhood (15,16). We report the first case of CPP during rhGH replacement in a male patient with PWS without hypothalamic imaging abnormalities. Although it was suggested that one of the causes of precocious puberty or accelerated progression of puberty might be due to GH therapy associated with hypothalamic dysfunction, no clear causal association between the rhGH replacement and the CPP development has been made in this case. In conclusion, we would like to emphasize that CPP may be diagnosed in PWS patients, and the combined therapy (rhGH replacement and GnRHa) may be beneficial, since extreme short stature was avoided in this case, while appropriated pubertal progression was restored. Although the benefits of rhGH therapy in PWS is well established (2), there is no consensus in the management of such a gonadotrophic disorder because few cases of precocious puberty in patients with PWS have been reported in the literature.
